# Biomechanical Evaluation of Mitral Valve Repair: Virtual Chordal Transposition to Restore Anterior Leaflet Prolapse

**DOI:** 10.31083/j.rcm2412367

**Published:** 2023-12-26

**Authors:** Soohwan Jeong, Seong-Min Kim, Woojae Hong, Minsung Ko, David D. McPherson, Hyunggun Kim

**Affiliations:** ^1^Department of Biomechatronic Engineering, Sungkyunkwan University, 16419 Suwon, Gyeonggi, Republic of Korea; ^2^Division of Cardiovascular Medicine, Department of Internal Medicine, The University of Texas Health Science Center at Houston, Houston, TX 77030, USA

**Keywords:** mitral valve, chordal transposition, biomechanical, simulation, mitral valve repair

## Abstract

**Background:**

Surgical management of an anterior leaflet prolapse remains comparatively challenging and has led to the lack of any firmly established standard repair techniques, as seen in cases of posterior leaflet prolapse. Chordal transposition repair is widely acknowledged as a remarkably durable technique that utilizes the patient’s native chordae. This study aims to evaluate and predict the biomechanical and functional characteristics of a normal mitral valve (MV) model and a pathological MV model featuring anterior ruptured mitral chordae tendineae (RMCT), and to assess the effectiveness of the chordal transposition repair in the pathological MV model.

**Methods:**

There are four stages in the proposed virtual MV repair evaluation protocol: (1) modeling the virtual pathological MV model with an anterior (A2) RMCT; (2) performing chordal transposition as the virtual MV repair procedure; (3) dynamic finite element simulation of the normal (control) MV model, the pre-repair (pathological) MV model, and the post-repair (chorda transposition) MV model; (4) assessment and comparison of the physiological and biomechanical features among the normal, pre-repair, and post-repair cases.

**Results:**

The pathological MV model with anterior RMCT clearly demonstrated a substantial flail, a marked increase in chordal stresses on the two intact chordae adjacent to the ruptured A2 chordae, and severe anterior leaflet prolapse due to the A2 chordal rupture. The virtual chordal transposition demonstrated remarkable efficacy in mitigating the stress concentrations in the leaflet and chordae, restoring leaflet coaptation, and resolving anterior leaflet prolapse.

**Conclusions:**

This virtual MV surgery strategy offers a valuable means to predict, evaluate, and quantify functional and biomechanical improvements before and after MV repair, thereby empowering informed decision-making in the planning of chordal transposition interventions.

## 1. Introduction

Degenerative mitral valve (MV) disorder is characterized by distinct symptoms, including MV prolapse, which ranks among the most common MV anomalies. [1]. Degenerative MV disease commonly demonstrates a high correlation with mitral regurgitation (MR) and is characterized by gradual chordae rupture, chordae elongation, and annular dilation [2]. MR occurs when the MV fails to adequately close during left ventricular ejection into the aorta [3]. Posterior leaflet prolapse represents a prototypical example of degenerative MV disease, and cardiothoracic surgeons have developed diverse repair techniques to address posterior leaflet prolapse associated with MR, with several standard procedures becoming established [4]. Conversely, the surgical management of anterior leaflet prolapse remains comparatively challenging and has led to the lack of any firmly established standard repair techniques, as seen in cases of posterior leaflet prolapse [5, 6, 7].

Developed by Dr. Carpentier [8] in 1983, the chordal transposition repair technique was devised to correct MV prolapses. The sequential steps involved in this process are as follows [8, 9]: (1) the identification of the prolapsed segment; (2) selection of appropriate chordae and detachment from the posterior annulus; (3) excision and suturing of the leaflet in the chosen chordae region; (4) implantation of the excised chordae onto the free margin of the prolapsed leaflet. Chordal transposition repair is widely acknowledged as a remarkably durable technique that utilizes the patient’s native chordae instead of the neochordae [6, 9]. Moreover, when comparing various surgical treatment methods for chordae failure, including shortening, transposition, and replacement, the use of transposition demonstrates much higher freedom from recurrent MR compared to shortening (96 ± 2% vs. 74 ± 9%) [10]. Nonetheless, continuous research is essential given the extended duration of clinical studies and relatively longer surgical time associated with this approach, compared to other surgical techniques [6, 11].

In particular, there is evidence that chordal transposition shows better recovery for anterior leaflet prolapse compared to other surgical techniques [5, 12, 13, 14]. Nevertheless, the complexity of the surgical procedure and the prolonged duration of the operation contribute to relatively limited clinical outcomes when compared to quadrangular resection and neochordoplasty [6, 11, 13, 15, 16]. Notably, limited information exists regarding biomechanical comparisons of chordal transposition pre- and post-repair, which necessitates further investigation. We have previously established a comprehensive computational evaluation protocol encompassing techniques such as quadrangular leaflet resection and neochordoplasty [17, 18, 19, 20]. Simulations involving a computational MV model exhibiting ruptured mitral chordae tendineae (RMCT) offer valuable insights into the complex functional and biomechanical aspects of pathological MVs before and after repair [17, 18]. In addition, the assessment of biomechanical information relating to the anterior RMCT MV model before and after chordal transposition may provide robust supporting data that helps to establish standard surgical techniques for addressing anterior leaflet prolapse.

This study aims to evaluate and predict the physiological and biomechanical features of a normal MV model and a pathological MV model featuring anterior RMCT, and to assess the effectiveness of the post-chordal transposition repair MV model. Computational simulations employing dynamic finite element (FE) methods were conducted to compare the MV function before and after chordal transposition.

## 2. Materials and Methods

### 2.1 Virtual Chordal Transposition Repair Protocol

**Fig. 1.**
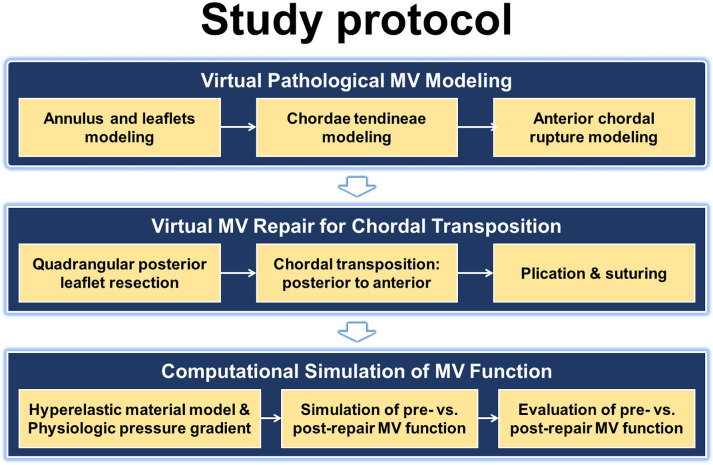
**Computational mitral valve (MV) repair evaluation protocol for virtual chordal transposition**.

The computational MV repair evaluation protocol employed in this study contains several key steps: (1) modeling the virtual pathological MV featuring anterior (A2) chordal rupture; (2) using chordal transposition to perform the virtual MV repair; (3) conducting dynamic FE evaluations of the normal (control) and pathological MVs before and after repair (chordal transposition); (4) assessing and comparing the physiological and biomechanical features among the normal, pre-repair, and post-repair models (Fig. 1). The virtual MV models were created using our in-house MV modeling code (MATLAB, MathWorks Inc, USA), and dynamic FE evaluations were conducted by ABAQUS (Version 2017, SIMULIA, Dassault Systems, Waltham, MA, USA).

### 2.2 Pathological MV Featuring A2 RMCT

**Fig. 2.**
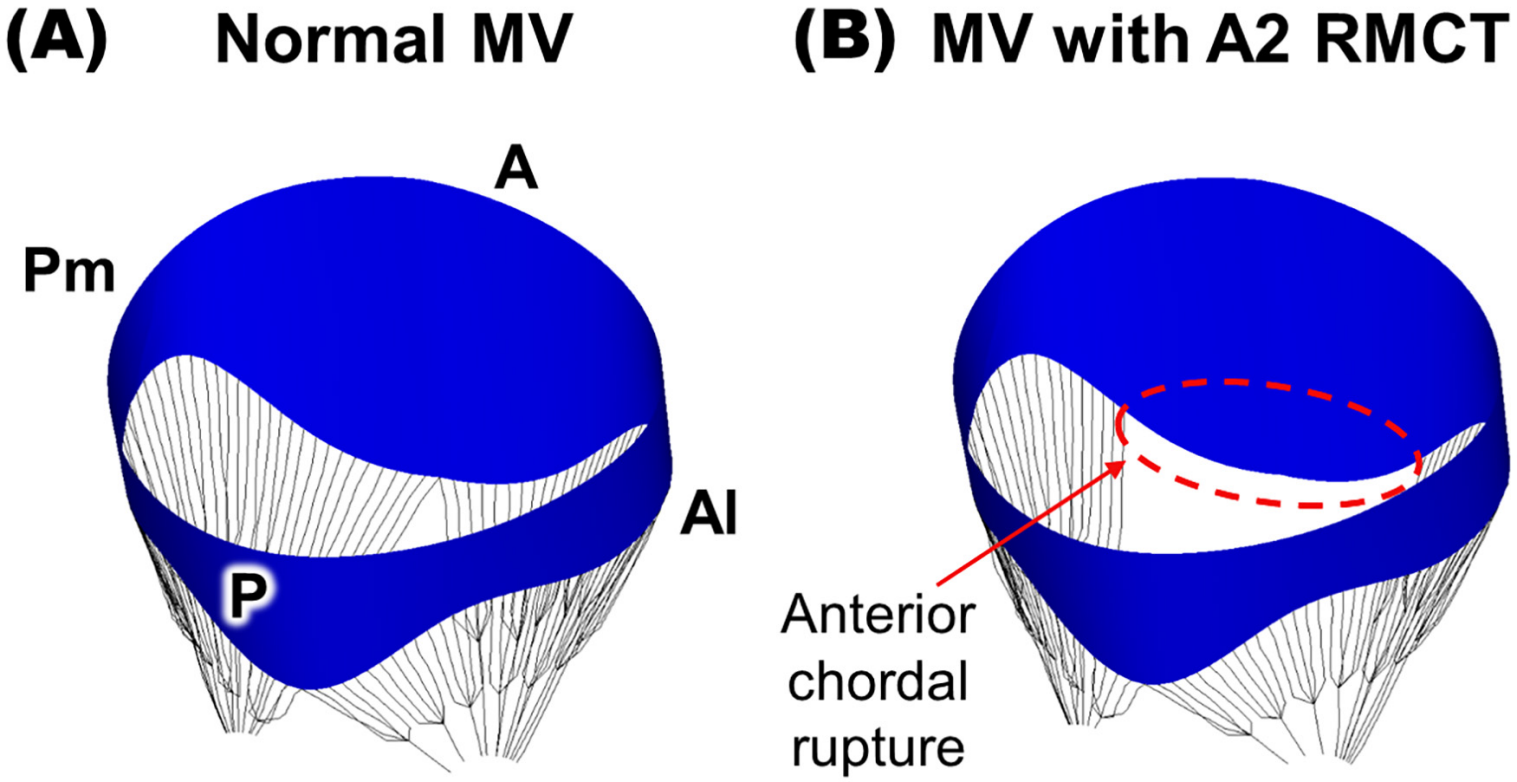
**Virtual mitral valve (MV) models**. (A) Normal MV, (B) MV with A2 ruptured mitral chordae tendineae (RMCT). Pm, posteromedial; A, anterior; Al, anterolateral; P, posterior.

A normal MV model near-end diastole consisted of a saddle-shaped annulus, a single-cusp anterior leaflet, a tri-scalloped posterior leaflet, and marginal and strut chordae tendineae (Fig. 2). The MV annulus was divided into anterior and posterior tracts, featuring a posteromedial–anterolateral distance of 32.0 mm and anterior–posterior distance of 31.5 mm [17, 18]. The leaflet-free margin was defined using a linear combination of sinusoidal functions, and multiple lines were generated connecting the annulus to the leaflet-free margin [21, 22]. A three-dimensional (3D) membrane structure of the MV leaflet was designed, which utilized a non-uniform rational B-spline surface (NURBS) modeling approach [19, 23], and exported to ABAQUS followed by meshing with 4122 triangular 3D shell elements. The normal MV model included 24 marginal chordae and 2 strut chordae. These components played a crucial role in linking the papillary muscles with both leaflets. Each marginal chordae tendineae extended into five marginal chordae, which effectively secured the leaflet along its entire free edge. The two strut chordae were modeled and demonstrated a distinct sequential arrangement pattern of papillary muscle tips, primarily localized to the central region of the anterior leaflet. All the chordae tendineae were represented as 3D line elements (T3D2 element type). In order to construct the pathological MV model with A2 RMCT, four primary chordae in the A2 scallop were selectively excised, as guided by previous clinical reports, resulting in the absence of chordae within the length range of 8–12 mm along the marginal edge of the leaflet [24].

### 2.3 Virtual MV Repair: Chordal Transposition

**Fig. 3.**
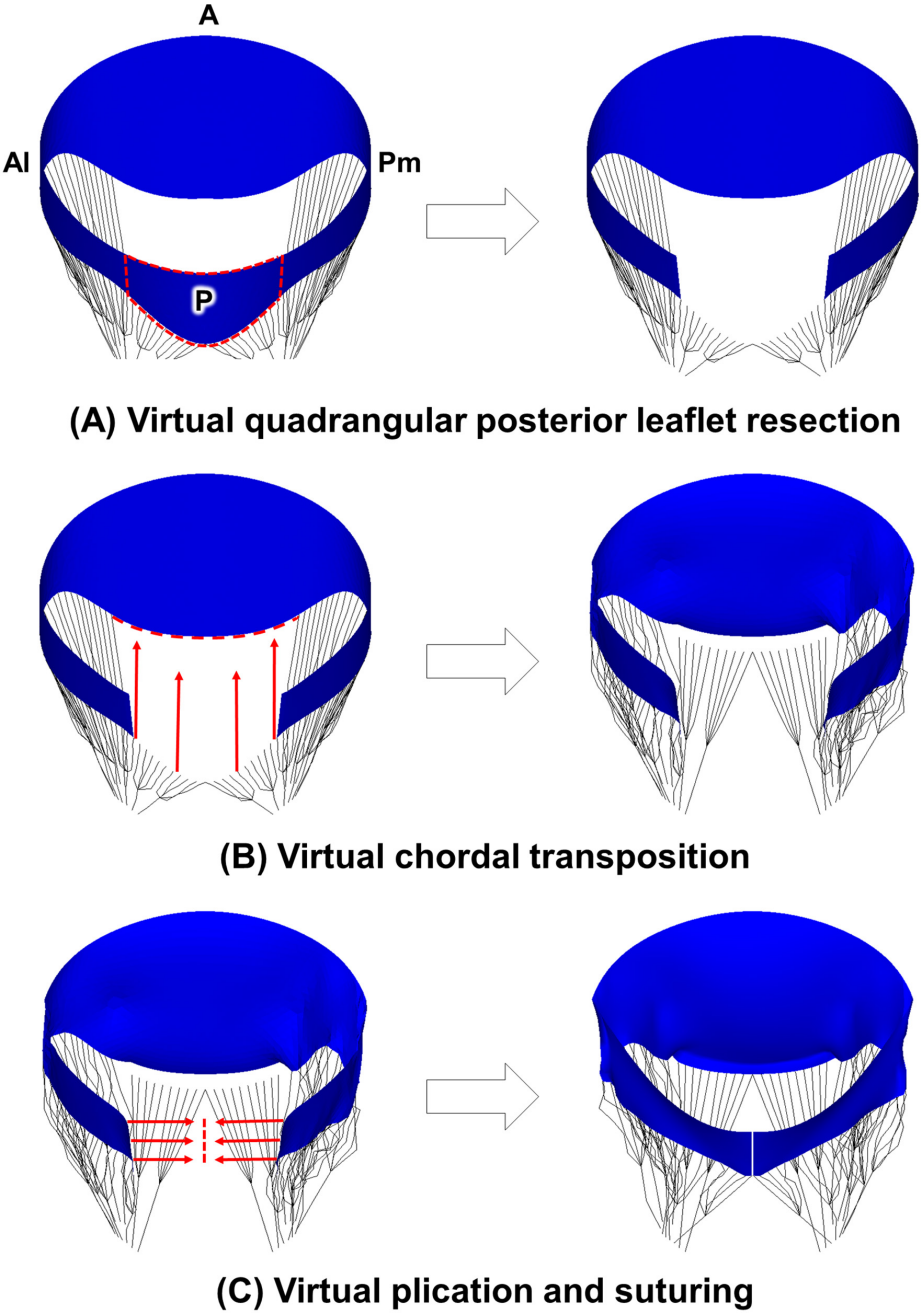
**Computational evaluation protocol for virtual chordal transposition repair**. (A) Virtual quadrangular posterior leaflet resection. (B) Virtual chordal transposition. (C) Virtual plication and suturing. Pm, posteromedial; A, anterior; Al, anterolateral; P, posterior.

Fig. 3 illustrates the step-by-step process of virtual chordal transposition repair. Initially, virtual quadrangular posterior resection is performed, targeting the posterior (P2) scallop, where the chordae tendineae being transferred to the A2 scallop are connected. A total of four primary marginal chordae were identified for the transfer process, necessitating a 16 mm resection of the P2 scallop, to which these chordae were attached. Subsequently, the detached chordae from the P2 scallop were relocated to the A2 scallop, ensuring alignment in the anterior-to-posterior direction. Then, the transferred chordae were integrated with the elements corresponding to the A2 leaflet free margin, thereby replicating the intricate process of chordae suturing. In the last step of the virtual chordal transposition, the P2 scallop was subjected to plication and suturing. Converging the two excised leaflet edges at the midpoint ensured uniform spacing, while interconnecting the corresponding elements in the leaflet tissue replicated the suturing effect.

### 2.4 FE Evaluations of MV Function

The identical dynamic FE simulation protocol, which has been rigorously demonstrated and validated in our previous studies, was employed in this study to perform the MV function simulations [17, 18, 19, 20, 23, 25, 26]. Briefly, a Fung-type elastic constitutive model was utilized to accurately represent the anisotropic hyperelastic behavior of the MV leaflet tissue [18, 27, 28]. The relationship between the Green–Lagrange strain (*E*) and Cauchy stress (σ) was established as:

(1) $$\sigma =\frac{1}{J}\,𝐅\,\frac{\partial W}{\partial E}\,{𝐅}^{T}$$

where J represents the determinant of the deformation gradient (**F**). Moreover, to precisely capture the material characteristics of the leaflets, the strain energy function (*W*) was defined using four parameters (*c*, *A1*, *A2*, and *A3*) as follows.

(2) $$\begin{array}{c} W=\frac{c}{2}\,\left[e^{Q}-1\right],Q=A_{1}\,E_{11}^{2}+A_{2}\,E_{22}^{2}+2\,A_{3}\,E_{11}\,E_{22} \end{array}$$

the leaflet tissue elements were approximated as nearly incompressible and hyperelastic (J = det F = 1). In accordance, the stress–strain correlation was formulated as follows:

(3) $$\sigma_{c}=\left(2\,E_{11}+1\right)\,c\,\exp\left(Q\right)\,\left(A_{1}\,E_{11}+A_{3}\,E_{22}\right)$$

(4) $$\sigma_{r}=\left(2\,E_{22}+1\right)\,c\,\exp\left(Q\right)\,\left(A_{3}\,E_{11}+A_{2}\,E_{22}\right)$$

the primary material coordinates considered in our analysis were the fiber directions spanning the surface of the leaflet, namely, the circumferential (σ_c_) and radial (σ_r_) directions. To accurately capture the mechanical response of the leaflet tissue, we incorporated experimentally derived material parameters from a previous investigation [29] into the Fung-type hyperelastic constitutive model, which was then integrated into the ABAQUS platform. The anterior leaflet had a thickness of 0.69 mm, while the posterior leaflet had a thickness of 0.51 mm [30]. Next, the Ogden models were employed to characterize the nonlinear hyperelastic properties of the strut and marginal chordae [31]. The cross-sectional areas assigned to the strut, anterior marginal, and posterior marginal chordae were 0.61 ${\mathrm{mm}}^{2}$, 0.29 ${\mathrm{mm}}^{2}$, and 0.27 ${\mathrm{mm}}^{2}$, respectively [31]. To ensure accurate simulations, the virtual MV models were assigned appropriate values for Poisson’s ratio (0.48) and density (1100 kg/$m^{3}$) [23, 32, 33].

Subsequently, dynamic FE analyses were conducted to assess the MV functions before and after repair. A physiological transvalvular pressure gradient was applied to the simulations throughout an entire cardiac cycle (duration = 1.8 seconds, end-systolic pressure = 126 mmHg [34]). To accurately represent the coaptation between the two leaflets, self-contact within each leaflet, and the interaction between the leaflets and chordae during the cardiac cycle, the penalty method was utilized to implement the general contact algorithm (friction coefficient of 0.05) [26].

### 2.5 Assesment of Virtual Chordal Transposition

In order to assess the impact of the MV repair technique, an analysis of the leaflet stress distribution, the stress distribution along the chordae, and the coaptation between the leaflets was conducted across the entire cardiac cycle. Specifically, the biomechanical properties of the normal MV and the pathological MV before and after chordal transposition were compared during peak systole. Both quantitative and qualitative assessments were performed to evaluate the functional and morphological characteristics of these models. Geometric variables, including coaptation length and coaptation angle, were also examined. Coaptation length was defined as the distance from the free margin of the anterior leaflet to the highest point of the leaflet coaptation at peak systole. The coaptation angle was determined by connecting the top of the leaflet coaptation with the anteroposterior (A–P) line.

## 3. Results

### 3.1 Leaflet Stress Distributions

**Fig. 4.**
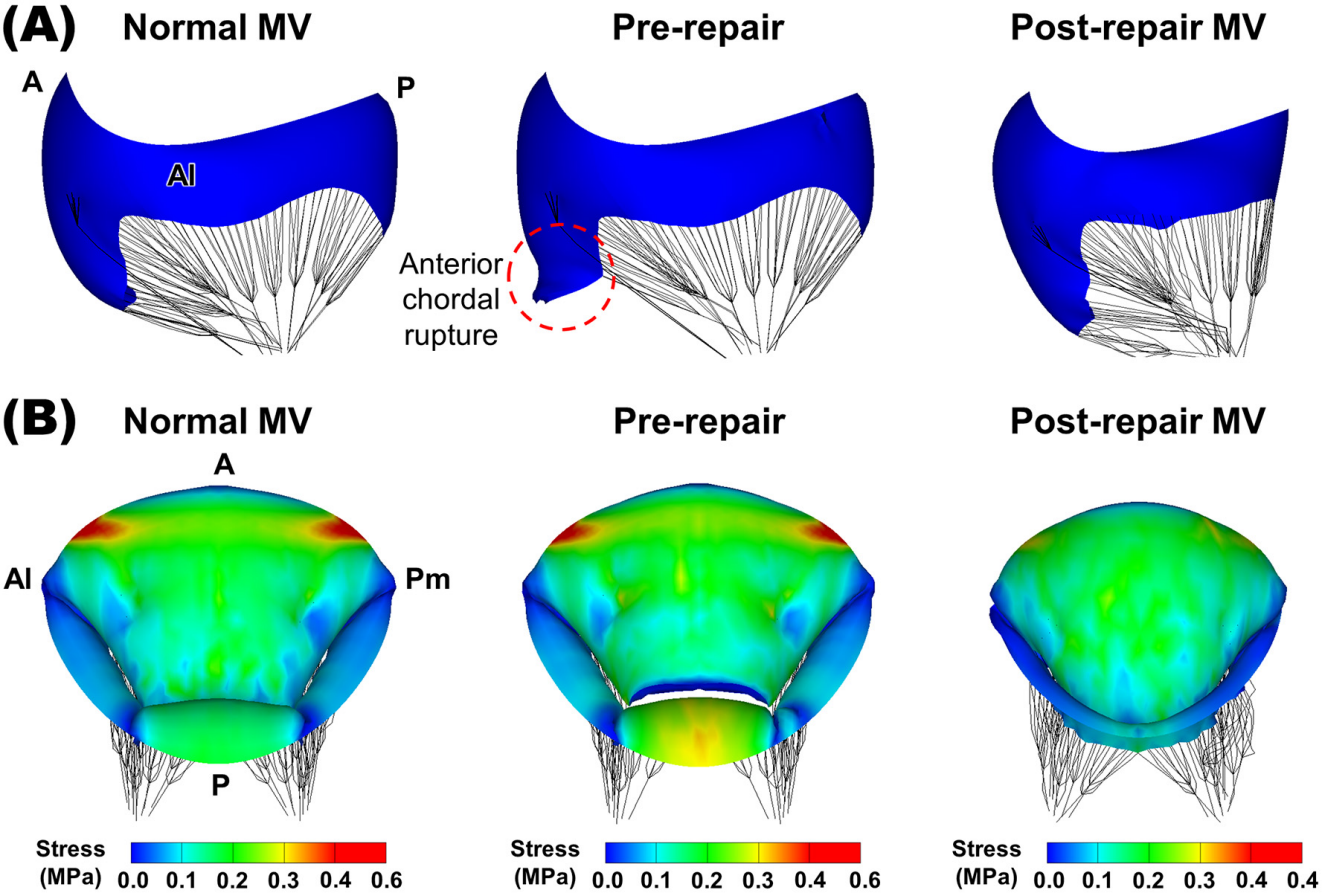
**MV morphologies and leaflet stress distributions**. (A) MV morphologies at end-diastole for the normal, pre-repair, and post-repair MV models. (B) Leaflet stress distributions near peak systole. Pm, posteromedial; A, anterior; Al, anterolateral; P, posterior; MV, mitral valve.

Successful simulation of virtual chordal transposition repair was carried out for the pathological MV model presenting an A2 chordal rupture. Fig. 4A illustrates the MV morphologies at end-diastole for the normal MV and the pathological MV before and after chordal transposition. The pathological MV model clearly exhibited A2 chordal rupturing and a substantial anterior leaflet flail (Fig. 4A). Conversely, the post-virtual chordal transposition MV model revealed a considerable reduction in the total annulus length, equivalent to the length of the excised P2 leaflet.

Fig. 4B displays the leaflet stress distributions near peak systole for the normal, pre-repair, and post-repair MV models. In order to facilitate a comparison among these models, a reference maximum stress value (0.4 MPa) was established to visualize the MV models [19]. In the normal MV, the notable stress values were observed in the circumferential direction within the anterior saddle-horn region, reaching a maximum stress of 0.6 MPa. The pathological MV model with an A2 chordal rupture exhibited a stress distribution resembling that of the normal MV in the anterior leaflet region, with a maximum stress of 0.61 MPa. However, the P2 leaflet region in the pre-repair MV displayed a relatively higher stress distribution compared to the normal MV. Additionally, following virtual chordae transposition repair, there was a substantial reduction in the overall stress throughout the leaflet region, effectively mitigating the high-stress distribution observed in the pre-repair P2 scallop. Moreover, the considerable stresses identified in the radial direction within the central zone of the anterior leaflet were effectively alleviated in both the normal and pathological MVs. The maximum leaflet stress value of 0.37 MPa was found after the virtual chordal transposition and fell below the designated threshold for maximum stress. This reduction indicates a substantial decrease of 60.6% in comparison to the maximum stress detected in the pathological MV.

### 3.2 Chordal Stress Distributions

**Fig. 5.**
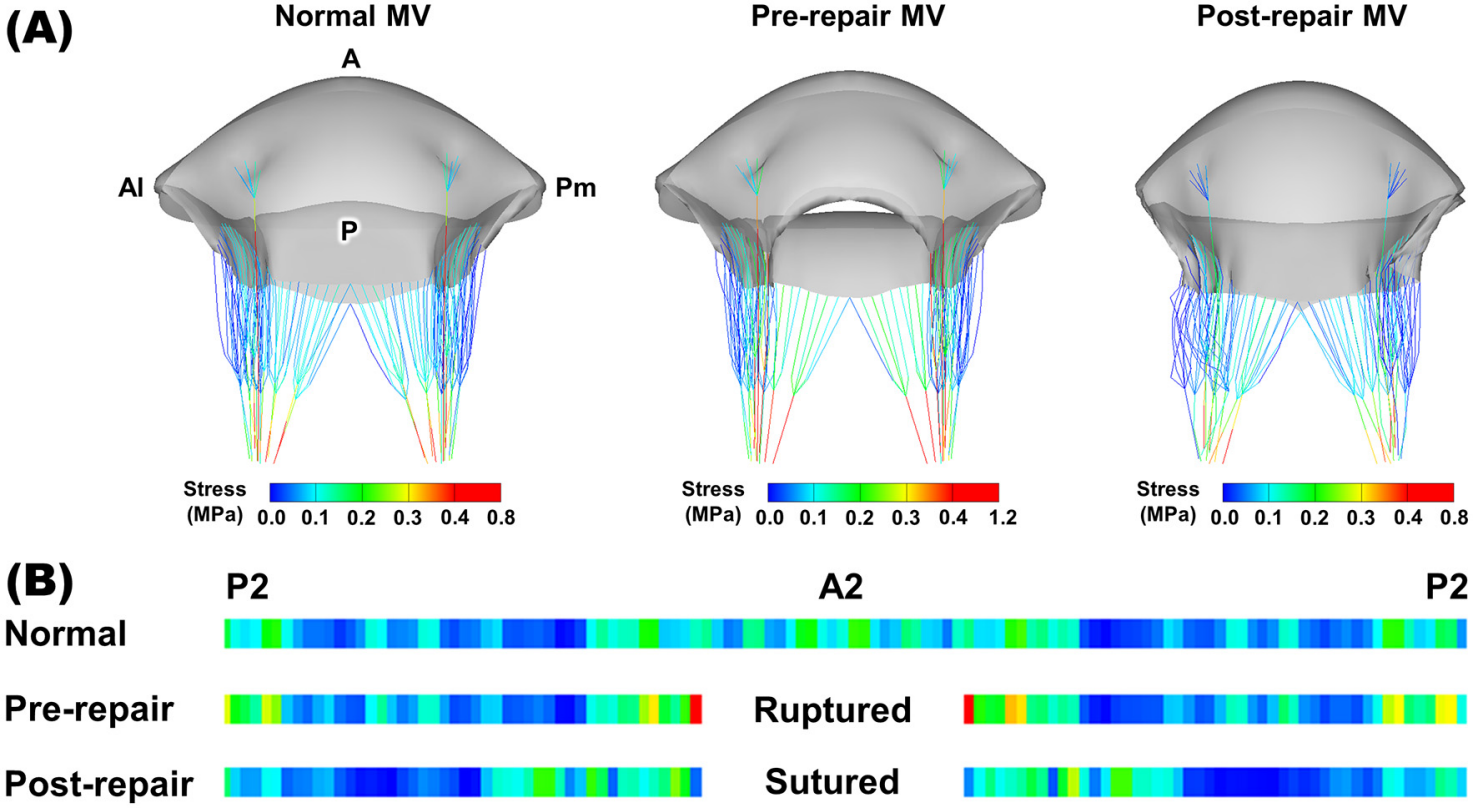
**Chordal stress distributions**. (A) Chordal stress distributions near peak systole for the normal, pre-repair, and post-repair MV models. (B) Unfolded chordal stress distributions. MV, mitral valve; Pm, posteromedial; A, anterior; Al, anterolateral; P, posterior.

During peak systole, the chordal stress distributions were assessed and compared among the normal MV and the pathological MV before and after chordal transposition (Fig. 5A). Fig. 5B displays the unfolded chordal stress distributions, which were centered on the A2 scallop and bisected at the midpoint of the P2 scallop. In the normal MV, relatively higher chordal stress values were primarily localized in the A2 and P2 leaflet regions rather than the entire chordae, while no abrupt stress variations were noted within the specific chordae. The maximum chordal stress value was 0.81 MPa in the normal MV. In contrast, the pre-repair MV featuring A2 RMCT demonstrated a marked increase in chordal stresses on the two intact chordae, adjacent to the ruptured A2 chordae, which reached a maximum chordal stress value of 1.17 MPa. The P2 region of the pathological MV showed a relatively higher stress distribution in comparison to the normal MV, while other areas displayed a comparable chordal stress distribution. Following the virtual chordal transposition repair procedure, the post-repair MV displayed a large reduction in the high chordal stress values within both the A2 and P2 leaflet regions, where the maximum stress value only reached 0.78 MPa, thereby indicating a lower chordal stress level compared to the normal MV. Particularly noteworthy were the dramatically decreased chordal stress values identified in the intact chordae that were located adjacent to the ruptured A2 chordae. The chordal stress distributions in the A2 and P2 leaflet regions of the post-repair MV demonstrated relatively uniform stress values without any large fluctuations, thereby resembling that of the normal MV.

### 3.3 Pre-Repair vs. Post-Repair Leaflet Coaptation

**Fig. 6.**
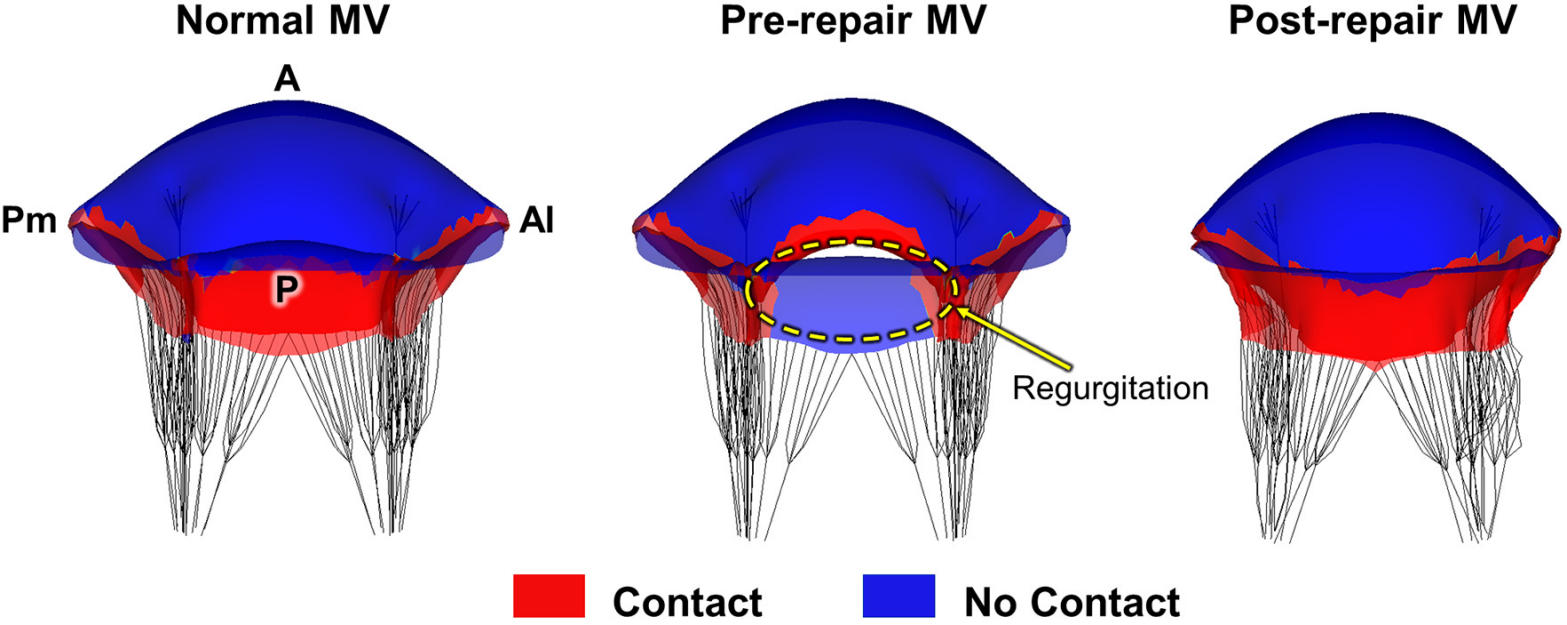
**Leaflet coaptation near peak systole for the normal, pre-repair, and post-repair MV models**. MV, mitral valve; Pm, posteromedial; A, anterior; Al, anterolateral; P, posterior.

Fig. 6 presents the visualization and identification of leaflet coaptation at peak systole, thereby enabling a qualitative comparison of the performance between the normal MV and the pathological MV, both before and after chordal transposition. The normal MV exhibited complete coaptation, while the pathological MV exhibited pronounced anterior leaflet prolapse resulting from the A2 chordal rupture, which was accompanied by a distinct area of MR. Remarkably, in the post-repair MV, successful restoration of the anterior leaflet prolapse and complete recovery of the coaptation were both clearly observed, which surpassed the coaptation performance by the normal MV. The virtual chordal transposition repair facilitated the junction between the two leaflets, effectively restoring the leaflet coaptation.

### 3.4 Coaptation Lengths and Coaptation Angles

**Fig. 7.**
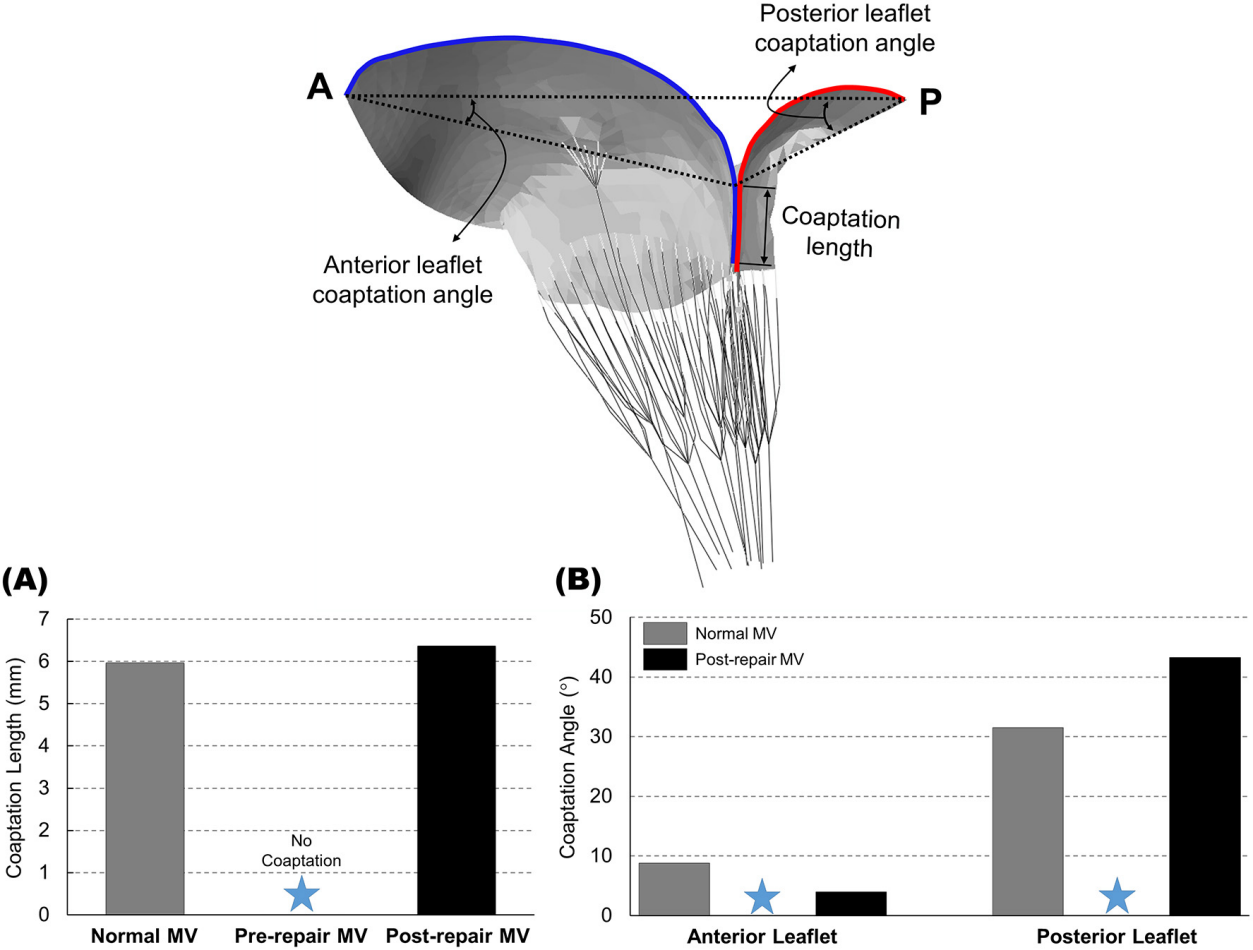
**(A) Coaptation lengths and (B) coaptation angles near peak systole for the normal, pre-repair, and post-repair MV models**. The cross-sectional edge of the anterior leaflet in the A–P plane is visualized in blue, while the posterior leaflet is in red. A, anterior; P, posterior; MV, mitral valve. Blue star, no coaptation.

Assessing the morphological attributes of the normal MV and the pathological MV, both before and after the chordal transposition, at the fully closed position enabled a strategic examination of the coaptation lengths and coaptation angles of the two leaflets (Fig. 7). For enhanced visualization and quantitative analysis of the leaflet coaptation length, the cross-sectional edges of the leaflets in the A2–P2 plane were color-coded, with the anterior leaflet depicted in blue and the posterior leaflet depicted in red. During peak systole, the normal MV showed a leaflet coaptation length of 5.97 mm, while no leaflet contact was observed in the pathological MV featuring A2 RMCT (Fig. 7A). Remarkably, the leaflet coaptation length of the repaired MV with chordal transposition was completely restored and measured 6.37 mm. Furthermore, the post-repair MV demonstrated a reduced anterior leaflet coaptation angle of 4.0°, in comparison to the normal MV (8.8°), whereas the posterior leaflet coaptation angle increased to 43.3° in the post-repair MV, surpassing the normal MV (31.5°) (Fig. 7B). These findings provide evidence that chordal transposition repair effectively eliminated the severe anterior leaflet prolapse.

## 4. Discussion

The current standard clinical protocol for the management and evaluation of MV pathology involves the utilization of transthoracic echocardiography (TTE) and transesophageal echocardiography (TEE). Modern advancements in 3D TEE technology have greatly enhanced the assessment of the volumetric morphology of the pathological MV apparatus and the examination of specific flow characteristics, such as the MR jet passing through the MV leaflets [17]. Biomechanical information, including abnormal or highly-elevated leaflet stresses, excessive chordae stress, and the detailed distribution of 3D leaflet coaptation, can yield valuable insights for comparing, determining, and improving the effectiveness of various repair techniques in restoring normal MV function. Nevertheless, in order to obtain these comprehensive biomechanical insights, synergistic integration of computational simulation and patient-specific 3D TEE data is required [18, 19, 35, 36, 37].

The occurrence of MV prolapse can be attributed to an excess of leaflet tissue in younger patients, whereas in the elderly population, fibroelastic deficiency is the primary etiological factor [1]. These conditions contribute to the development of degenerative MV pathologies, which are characterized by leaflet prolapse, chordal rupturing, chordal elongation, and subsequent MR [38]. MVs with RMCT are particularly susceptible to pathological tissue proliferation, which can lead to severe prolapse or additional chordae rupturing over time [39]. Therefore, the principal aim of surgical intervention for cases of RMCT is to restore the leaflet coaptation and normalize the structure and function of the MV [2, 38]. Various surgical techniques have been developed and evaluated for MV repair in clinical studies, including ring annuloplasty, leaflet resection, neochordoplasty, edge-to-edge repair, and chordal transposition. The quadrangular leaflet resection technique has been established as the standard approach for repairing prolapsed MVs [40]. Additionally, neochordoplasty using pre-measured expanded polytetrafluoroethylene (ePTFE) neochordae has been introduced as an alternative standard approach to restore MV function without the need for leaflet tissue excision [41]. Despite the establishment of these standard techniques for repairing RMCT on the posterior leaflet, the absence of a corresponding MV repair technique for the anterior leaflet RMCT remains a prevailing issue [5, 7].

The present study aimed to computationally evaluate the physiological and biomechanical properties of a normal MV model and a pathological MV model featuring an anterior RMCT. Furthermore, the study aimed to assess the effectiveness of virtual chordal transposition in repairing an MV model with an anterior RMCT. Following the virtual chordal transposition repair, substantial alterations in the biomechanical properties of the MV were observed in comparison to the pathological MV with an A2 chordal rupture. Excision of the P2 scallop region resulted in morphological alterations, including a reduction along the annulus area. These morphological alterations exerted a significant influence on the biomechanical properties of the MV apparatus and the physiological aspects of the MV function following chordal transposition (Fig. 4). In the post-repair MV, a distinct recovery in stress distribution within the P2 leaflet area was observed. While stress concentration was relatively prominent in the anterior region due to a reduction in the posterior leaflet area, induced by quadrangular resection, the overall stress distribution in the post-repair MV was much lower compared to both the normal and pathological MVs. Interestingly, the high-stress concentration near the aortic–mitral curtain area in the anterior leaflet was eliminated. Furthermore, a noticeable redistribution of chordal stress distribution was observed after virtual chordal transposition repair (Fig. 5). In the pathological MV, excessively elevated stresses were observed in the remaining neighbor chordae adjacent to the ruptured A2 chordae. However, after virtual chordal transposition, these stresses underwent a dramatic reduction, reverting to normal levels. This indicates the successful transfer and suturing of the remaining chordae from the resected P2 scallop to the A2 scallop, thereby achieving the restoration of the A2–P2 leaflet coaptation and the recovery of normal chordal stresses. Complete leaflet coaptation was achieved following virtual chordal transposition repair (Fig. 6). During peak systole, the pre-repair MV exhibited a lack of leaflet coaptation and concomitant MR in the A2–P2 region. In contrast, the post-repair MV demonstrated a fully restored leaflet coaptation, which was comparable to that of the normal MV. These findings substantiate the fundamental objective of surgical interventions, which aim to reinstate the normal MV function [42]. Quantitative assessment of leaflet coaptation in all the MV models involved measuring coaptation lengths and coaptation angles (Fig. 7). The pre-repair MV featuring A2 RMCT demonstrated a complete absence of coaptation length on the A–P line, whereas the post-repair MV displayed a fully restored coaptation length (6.37 mm) that was comparable to that of the normal MV (5.97 mm). After virtual chordal transposition repair, the coaptation angle of the posterior leaflet increased, while that of the anterior leaflet decreased, compared to the normal MV. This discrepancy in coaptation angles arose from the posterior displacement of the leaflet coaptation location, which was attributable to the resection of the posterior leaflet, and in contrast to the normal MV. These findings provide compelling evidence of the quantitative restoration of MV function being achieved through virtual chordal transposition repair, while also corroborating earlier clinical investigations [5, 14].

This study encompasses several limitations and simplifications that should be carefully acknowledged, thereby advising against direct extrapolation of the findings to clinical scenarios. Despite the fact that both the mechanical properties and physical dimensions (e.g., tissue thickness) of the pathological MV leaflets and chordae tendineae are different from healthy MV tissue, we employed the same mechanical property for all the MV models. This simplification was employed to prioritize the comparison of the effects of the chordal transposition on the biomechanical and physiologic characteristics of MV function. The simulation outcomes may differ if actual human tissue data are incorporated and should be carefully interpreted in a qualitative and comparative manner. Our investigation employed a normal MV model and a pathological MV model involving A2 RMCT, both featuring parametric designs intended to be symmetrically aligned with the posteromedial–anterolateral direction. While this parametric MV model facilitates the assessment of specific designs, it is essential to note that actual patient MVs with an A2 RMCT and their post-repair MVs may exhibit diverse physiological and biomechanical characteristics. To fundamentally address this concern, we are currently collecting patient-specific 3D TEE and tissue data from patients having A2 RMCT, both before and after chordal transposition repair, to allow for computational evaluations within the framework of individual MV surgical cases. While ring annuloplasty is typically performed in conjunction with chordal transposition repair to enhance surgical durability and annular stability [9, 43], ring annuloplasty was intentionally excluded from our study design, thereby constituting an essential design factor. By focusing solely on chordal transposition, we aimed to avoid confounding influences from other interventional techniques and to assess the biomechanical and physiological efficacy of chordal transposition in the repair of anterior RMCT. Consequently, repair parameters, such as the shape, location, and dimensions of leaflet resection, along with the extent and location of the chordal transfer with or without the sliding technique, were not explicitly explored in this study. The application of meticulous computational evaluations, integrating patient-specific repair parameters, holds great potential in optimizing pre-operative planning for MV repair. These rigorous evaluations enhance the accuracy of computational predictions, thereby providing valuable insights into the expected post-repair outcomes.

## 5. Conclusions

In the present study, a new virtual MV repair evaluation protocol was demonstrated, allowing for the assessment and prediction of MV function both before and after chordal transposition repair. Our virtual chordal transposition technique demonstrated remarkable efficacy in mitigating the stress concentration in the leaflet and chordae, restoring leaflet coaptation, and resolving anterior leaflet prolapse. The achieved level of biomechanical similarity to a normal MV was highly commendable, with the exception of any anticipated morphological changes resulting from the posterior leaflet resection. This strategy offers a valuable means to predict, evaluate, and quantify functional and biomechanical improvements before and after MV repair, thereby empowering informed decision-making in the planning of chordal transposition interventions.

## Data Availability

The datasets used and/or analyzed during the current study are available from the corresponding author on reasonable request.

## Abbreviations

3D, three-dimensional; FE, finite element; MR, mitral regurgitation; MV, mitral valve; RMCT, ruptured mitral chordae tendineae; TEE, transesophageal echocardiography; TTE, transthoracic echocardiography.
